# Supplementary material for: Content-rich biological network constructed by mining PubMed abstracts
Source: BMC Bioinformatics. 2004 Oct 8;5:147. doi: 10.1186/1471-2105-5-147 (PMC528731; doi:10.1186/1471-2105-5-147)
Supplement: Additional File 5 — The original Chilibot query results of the term "long-term potentiation (LTP)" and 22 other terms, limiting the latest references analyzed to the years 1990, 1995, 2000, and 2004. [file 1471-2105-5-147-S5.bz2 › chilibotAdditionalFile5/ltp1995/html/PKA_NMDA.html]

 


 **PKA** and **NMDA** 
  
Found 13 abstracts in PubMed,  **13 abstracts were retrieved and analyzed**.  


---

 Search Google  |
 PDF files only 
|  EDU domain only 

---

**Interactive relationship** (e.g. stimulation, inhibition, etc)

- Single channel analysis revealed that an adenosine monophosphate dependent protein kinase  **PKA**  increases the opening frequency and the mean open time of the non  **NMDA**  type glutamate receptor channels.  Ref: 1716001 Science, 1991
- Whole cell current response to  **NMDA**  was enhanced by 8 Br cAMP, a membrane permeant analog of cAMP or by intracellular application of cAMP or catalytic subunit of  **PKA** .  Ref: 8272253 Neurosci Lett, 1993
- However, the  **PKA**  antagonist Rp adenosine monophosphothioate Rp cAMPS did not alter the  **NMDA**  D serine response.  Ref: 8075826 Eur J Neurosci, 1994

**Parallel relationship** (e.g. studied together, co-existance, homology, etc.)

- To investigate the involvement of N methyl D aspartate  **NMDA**  receptor, protein kinase C PKC and calmodulin on long term potentiation LTP formation in the superior colliculus SC, the effects of an  **NMDA**  receptor antagonist D APV, PKC inhibitors H 7, K 252a, K 252b, polymyxin B, a protein kinase A  **PKA**  inhibitor H 8 and a calcium calmodulin dependent kinase inhibitor calmidazolium on LTP formation were studied in guinea pig SC slices.  Ref: 8097659 Brain Res, 1993
- The present study investigates the roles of protein kinase C PKC and A  **PKA**  activities in  **NMDA**  mediated calcium entry in primary cultures of mouse striatal neurons.  Ref: 8075826 Eur J Neurosci, 1994
- In this study 32P labeling of non  **NMDA**  GluRs was investigated in cultured hippocampal neurons stimulated 2 15 min with agonists that selectively stimulate either calcium calmodulin dependent protein kinase II CaM kinase II, calcium phospholipid dependent protein kinase C PKC, or cAMP dependent protein kinase A  **PKA** .  Ref: 7509863 J Neurosci, 1994
- Whole cell voltage clamp recording mode was used to examine the effect of adenosine monophosphate cAMP dependent protein kinase  **PKA**  on the responses of DH neurons to  **NMDA** .  Ref: 8272253 Neurosci Lett, 1993
